# Quantitative Measures of Time to Loss of 15% Vital Capacity and Survival Extension in Slowly Progressive Amyotrophic Lateral Sclerosis (ALS) Patients Treated with the Immune Regulator NP001 Suggests an Immunopathogenic Subset of ALS

**DOI:** 10.3390/biomedicines13123060

**Published:** 2025-12-12

**Authors:** Namita A. Goyal, Jinsy A. Andrews, Björn E. Oskarsson, Martina H. Wiedau, Edward J. Kasarskis, Bruce D. Forrest, Rongzhen Zhang, Paige M. Bracci, Matthew W. Davis, Ari Azhir, Michael S. McGrath

**Affiliations:** 1 Department of Neurology, University of California Irvine, Irvine, CA 92697, USA; 2 The Neurological Institute, Columbia University, New York, NY 10032, USA; 3 Department of Neurology, Mayo Clinic, Jacksonville, FL 32224, USA; 4 Department of Neurology, University of California Los Angeles, Los Angeles, CA 90095, USA; 5 Department of Neurology, University of Kentucky, Lexington, KY 40506, USA; 6 Hudson Innovations, LLC, Nyack, NY 10960, USA; 7 Department of Medicine, University of California San Francisco, San Francisco, CA 94110, USA; 8 Department of Epidemiology and Biostatistics, University of California San Francisco, San Francisco, CA 94158, USA; 9 Neuvivo, Inc., Palo Alto, CA 94301, USA

**Keywords:** ALS, predicted vital capacity (PVC), time to event (TTE), biomarker, slowly progressive ALS, disease progression rate (DPR), overall survival (OS), NP001

## Abstract

**Background/Objectives**: Overall survival in patients with amyotrophic lateral sclerosis (ALS) is linked to the rate of predicted respiratory vital capacity (PVC) loss. The objective of this study was to test whether changes in quantitative PVC measures over time linked to survival would define an immunopathogenic subset of ALS responsive to NP001, a regulator of innate immunity. **Methods**: In a retrospective study, data from intent-to-treat (ITT) population of two phase 2 trials of NP001 were evaluated for over time changes in PVC, time-to-event (TTE) loss of 15% PVC and PVC change from baseline, as linked to survival outcomes in patients treated with NP001 vs placebo. **Results**: Treatment with NP001 was associated with a significantly lower risk compared to placebo in the loss of 15% PVC over six months (*p* = 0.01; HR = 0.60, 95% CI: 0.39, 0.90). Data from the two trials were subsequently divided by a disease progression rate (DPR) value of 0.50 units of ALSFRS-R score lost per month for analysis of slow vs. rapid disease. In ALS patients with slowly progressive disease (DPR < 0.50), TTE PVC changes from baseline were slowed (*p* < 0.0005) and overall survival extended significantly (18.5 months) in NP001-treated vs. placebo groups. The rapidly progressive ALS patients (DPR ≥ 0.50) treated with NP001 showed no significant difference in PVC change or survival from the placebo group. **Conclusions**: These hypothesis-generating observations suggest that inflammation might play a significant role in the loss of respiratory function in a major subset of ALS patients.

## 1. Introduction

Monocyte activation is a prominent feature in the blood of patients with amyotrophic lateral sclerosis (ALS) [1]. NP001 (sodium chlorite) is a prodrug of the endogenous immunomodulator taurine chloramine [2]. Chlorite is converted to taurine chloramine (TauCl) within inflammatory monocytes and macrophages [3]. TauCl is well known as an innate immune regulator, proven to re-establish immune balance after inflammatory stimuli [4,5,6]. A phase 1 placebo-controlled trial of NP001 in patients with ALS demonstrated the safety, tolerability, and dose-dependent down-regulation of monocyte activation after a single dose [7]. The treatment effect of NP001, as measured using the Revised ALS Functional Rating Scale (ALSFRS-R) in two subsequent randomized phase 2 clinical trials in patients with ALS, was inconclusive [8,9]. However, a post hoc analysis of the pooled data from both trials demonstrated multiple effects on the innate immune system and preservation of vital capacity [2,9,10,11]. These hypothesis-generating observations led to the theory that since NP001 preserved vital capacity, it would also affect overall survival [12]. An analysis of overall survival (OS) was conducted using combined data from both Phase 2 NP001 studies. Data were collected and analyzed by an independent statistician under blinded conditions to minimize bias. The vital statuses of the intention-to-treat (ITT) participants who had received at least one dose of drug or placebo were obtained for OS analysis as described in Forrest’s report [13]. OS was longer in the NP001-treated group than in the placebo group in the ITT population; NP001-treated patients had a 23% lower risk of death than placebo-treated patients over the 10-year follow-up [13].

As ventilatory failure is the major cause of mortality in patients with ALS, the use of vital capacity (VC) is increasingly used as an objective biomarker of ALS progression [12,14]. In a comprehensive study of VC linked to survival, ALS patients ≤ 65 had an average loss of 15% of VC over six months, and these changes were related to OS [12]. In 2014, the FDA based the approvals of both nintedanib and pirfenidone on their demonstrated efficacy in slowing the decline of forced vital capacity (FVC) in patients with idiopathic pulmonary fibrosis (IPF) [15,16,17]. In 2018, in its ALS Guidance to Industry, the FDA specifically stated that FVC was an acceptable endpoint for evaluating treatment effectiveness in ALS and also recommended combining survival and function into a single overall measure (such as the joint rank test) [18].

Conventional drug developmental strategies typically target patients with rapidly progressive disease. The clinical trials of NP001 with ALS patients enrolled patients with symptom onset up to three years before initiation, therefore, including both slowly and rapidly progressive patients [8,9]. Importantly, recent ALS natural history studies have suggested that a slower form of the disease may be related to an immunologic state characterized as a chronic acute phase reaction [19,20,21].

In the current study, changes in vital capacity over time were assessed using a novel time-to-event (TTE) analysis. The TTE was defined as the time taken to lose 15% of predicted VC (PVC) from baseline values (the average lost over 6 months [12]) in the NP001-treated vs. placebo groups using the ITT data from two NP001 double blind placebo-controlled trials. These studies were also performed on data from patients with disease progression rate (DPR) values above and below 0.50 units of ALSFRS-R lost/month to separate them into slowly or rapidly progressive groups [7,22]. Conventional measures of VC loss, TTE loss of 15% PVC, and overall survival were determined in slow vs. rapidly progressive ALS subsets.

## 2. Materials and Methods

### 2.1. Clinical Trials and Data

Two NP001 phase 2 trials were conducted by Neuraltus Pharmaceuticals, Inc. (Palo Alto, CA, USA) in ALS patients and were registered on ClinicalTrials.gov (phase 2A: NCT01281631, 28 February 2011, and phase 2B: NCT02794857, 29 August 2016). These were both placebo-controlled 6-month studies. Phase 2A was completed in 2012 [8], and phase 2B in 2017 [9]. Both phase 2A and 2B studies were approved by the clinical site’s institutional ethics committees, and informed consent was obtained from all participants. Details of these two six-month trials have been published [8,9].

Complete files of data from the two phase 2 trials were evaluated as previously described [8,9,13], which also included the details of baseline demographics and characteristics of the patients receiving NP001 (sodium chlorite, Neuvivo Inc., Palo Alto, CA, USA) vs. placebo in the two trials that were utilized in the analysis of the intention-to-treat (ITT) population. The dosage of NP001 used in both clinical trials was 2 mg/kg body weight as chlorite (equivalent to 2.682 mg/kg sodium chlorite).

### 2.2. Definition of Analytical Clinical Factors

#### 2.2.1. Analysis of Predicted Vital Capacity (PVC) Change from Baseline

In the NP001 phase 2 trials, the phase 2A trial assessed the PVC using forced vital capacity (FVC), whereas the phase 2B assessed the PVC using slow vital capacity (SVC). Since PVC values between FVC and SVC are comparable [14], the evaluation of the effects of NP001 on the PVC change from baseline for both trials was performed using the formula below:

PVC change from baseline (%) = PVC at each timepoint (%) − PVC at baseline (%).

#### 2.2.2. Definition of ALS Disease Progression Rate (DPR) and Slow vs. Rapid ALS Groups

DPR was defined by the unit of ALSFRS-R score lost per month. Baseline DPR was calculated as follows:

(48 − ALSFRS-R score at baseline)/Duration of ALS symptom (months).

Slowly and rapidly progressive ALS were divided using the DPR at baseline: baseline DPR < 0.50 units of ALSFRS-R score lost/month was defined as slowly progressive ALS, and rapidly progressive ALS included those with a DPR ≥ 0.50 units of ALSFRS-R score lost/month at baseline [7,22].

### 2.3. Time-to-Event (TTE) Analyses of 15% PVC Loss

In the current study, we focused on the TTE analysis of PVC change from baseline in patients treated with 2 mg/kg of NP001 chlorite or placebo in the ITT population, which included all randomized subjects who received at least one dose of study infusion. The TTE evaluation of ≥15% PVC change was defined as the time in days from the date of randomization to the date when the %PVC loss reached 15% PVC, or to the last contact/known %PVC change < 15% for patients (censored) who were assessed over the 6-month trials.

### 2.4. Overall Survival (OS) Analyses

Survival data for ALS patients in the ITT population were obtained from Neuvivo Inc., and data were assessed through 30 September 2022. The detailed methods of OS analysis have been described in a recent report by Forrest et al. [13] and Zhang et al. [11].

### 2.5. Statistical Analyses

Statistical analysis was performed using JMP Student Edition 18 (SAS Institute, Cary, NC, USA) and packages “survival 3.8-3” [23] and “survminer 0.5.0” [24] in R 4.5.0 [25]. Data were summarized as counts and percentages for categorical data, using standard univariate descriptive statistics (number of participants, mean, standard deviation, median) for continuous/discrete data by treatment group. Preliminary analyses of categorical data were performed using Fisher’s exact test and Chi-square tests, and continuous/discrete data were analyzed using Wilcoxon rank sum tests.

TTE of PVC change and OS differences by treatment/group were assessed using a log-rank test. Kaplan–Meier methods were used to estimate no 15% PVC loss and survival probabilities and curves. Cox-proportional hazards models were used to estimate the hazard ratio associated with ≥ 15% PVC loss or dying for patients treated with NP001 compared with those given a placebo. The assumptions of proportionality of hazards over time were assessed using the Schoenfeld test. Patients who did not lose 15% PVC over the 6-month trial period were considered censored in the %PVC change TTE analyses.

All statistical tests were two-sided and considered statistically significant for a *p*-value < 0.05.

## 3. Results

### 3.1. Analysis of NP001 Activity Defined by Time-to-Event (TTE) Loss of 15% PVC

A total of 110 patients received a placebo, and 114 received 2 mg/kg NP001 in the ITT study. Patient demographic and clinical characteristics, including sex, age, site of ALS onset, El Escorial criteria, concurrent use of Riluzole, ALSFRS-R, duration of disease, and baseline PVC, did not differ by treatment groups [13].

**Figure 1:**
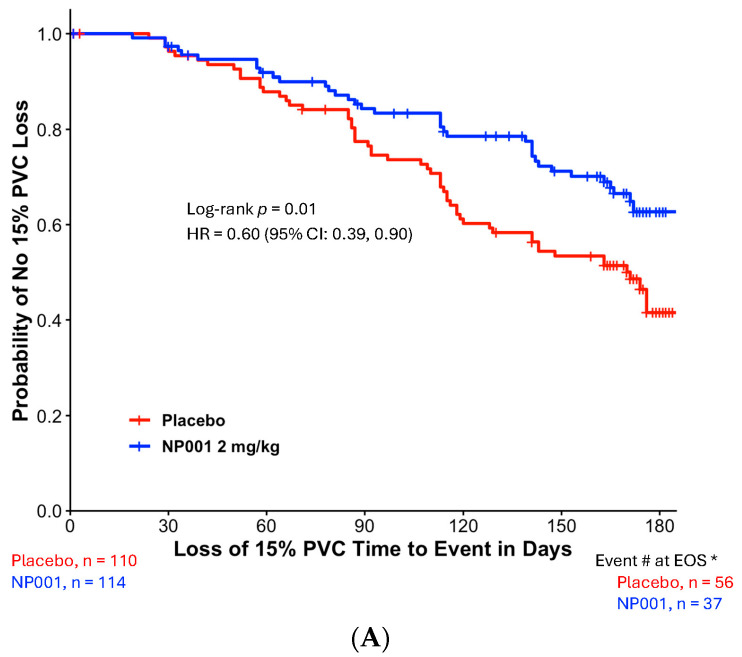

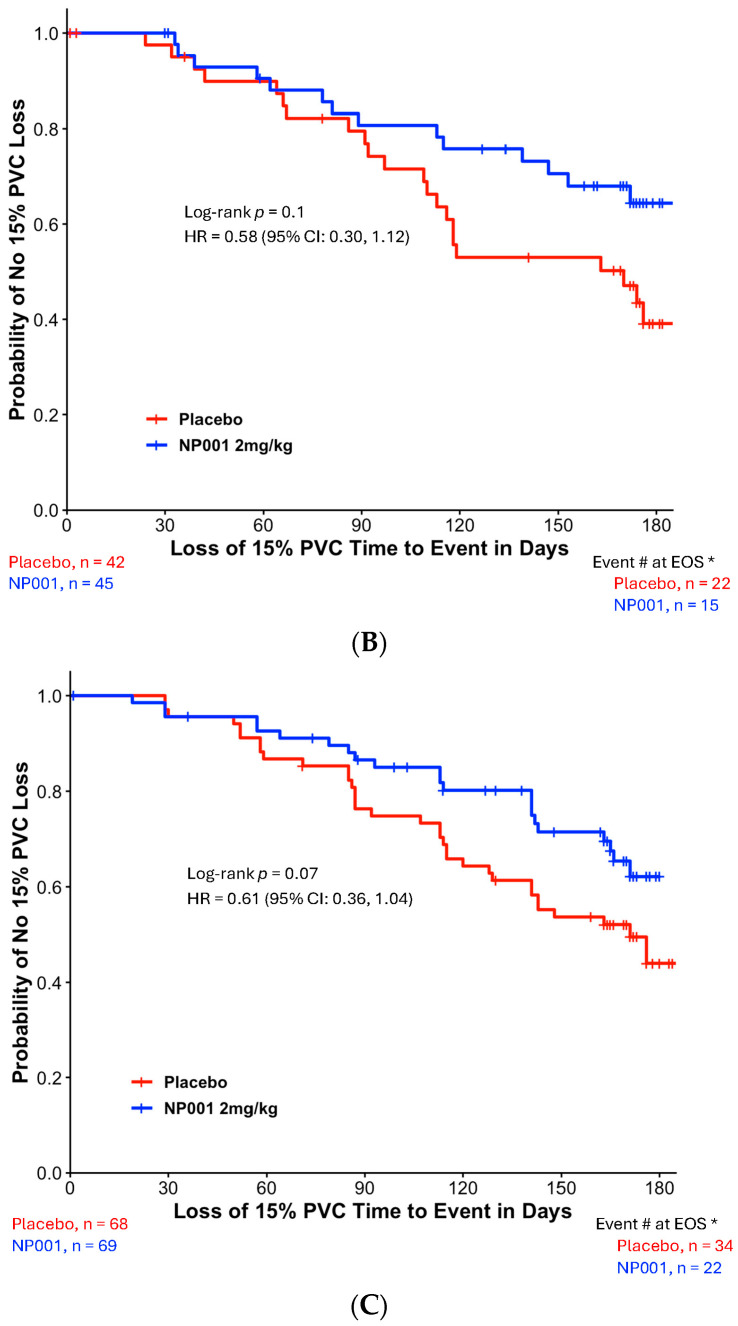
Kaplan–Meier plots of time to loss of 15% PVC in the NP001 phase 2 placebo-controlled clinical trials. All patients receiving a dose of the study drug were included in this intention-to-treat (ITT) analysis. The ‘event’ is an objective measure of treatment failure: the occurrence of a decline in PVC of ≥ 15% as an early sign of disease progression. Patients who did not reach the PVC endpoint were censored on the date of their last PVC assessment before or at the end of the 6-month period. *, Event # at EOS: total number of patients that had events (PVC decline of ≥15%) by the end of the study (EOS). (**A**) Kaplan–Meier plot of time to loss of 15% PVC from the pooled six-month phase 2 trials. ITT analysis in phase 2 with the 2 mg/kg NP001 group (blue, *n* = 114) and placebo (red, *n* = 110). Log-rank test, *p* = 0.01; HR = 0.60, 95% CI: 0.39, 0.90. (**B**) Kaplan–Meier plot of time to loss of 15% PVC from the phase 2A Trial. ITT analysis in phase 2A with the 2 mg/kg NP001 subgroup (blue, *n* = 45) and placebo (red, *n* = 42). Log-rank test, *p* = 0.1; HR = 0.58 (95% CI: 0.30, 1.12). (**C**) Kaplan–Meier plot of time to loss of 15% PVC from the Phase 2B Trial. ITT analysis in phase 2B with the 2 mg/kg NP001 subgroup (blue, *n* = 69) and placebo (red, *n* = 68). Log-rank test, *p* = 0.07; HR = 0.61 (95% CI: 0.36, 1.04).

The time to 15% PVC loss between the NP001 2 mg/kg and placebo groups was statistically significant in the TTE analysis of the ITT population (Figure 1A). NP001 2 mg/kg treatment was associated with a significantly lower risk of treatment failure compared to placebo at 6 months (Log-rank test, *p* = 0.01 and Hazard ratio (HR) = 0.60, 95% CI: 0.39, 0.90).

To test the variability of this outcome, the same ITT analysis was performed on data from each trial separately. Figure 1B shows the Kaplan–Meier plot of time to loss of 15% PVC from the phase 2A trial (Log-rank test, *p* = 0.1 and HR = 0.58, 95% CI: 0.30, 1.12). Similar results were observed in the phase 2B Kaplan–Meier plot of time to loss of 15% PVC (Log-rank test, *p* = 0.07 and HR = 0.61, 95% CI: 0.36, 1.04).

### 3.2. Relationship Between 15% PVC Loss in Slowly vs. Rapidly Progressive ALS

To evaluate whether the PVC change over 6 months was related to the rate of ALS disease progression, a Kaplan–Meier analysis was performed among the NP001-treated and placebo participants separated by a DPR of 0.50 units of ALSFRS-R score lost/month. Those below 0.50 were slowly progressive, and those above were rapidly progressive ALS patients. In this ITT analysis, 41% of patients from the combined phase 2A and 2B trials were categorized as experiencing slowly progressive ALS. Table 1 shows the baseline demographics and characteristics of the different DPR groups. The rapidly progressive group showed an advanced ALS status, characterized by a significantly lower ALSFRS-R score (*p* < 0.0001) and were relatively earlier in their ALS symptom onset (*p* < 0.0001) with higher DPR (*p* < 0.0001).

**Figure 2:**
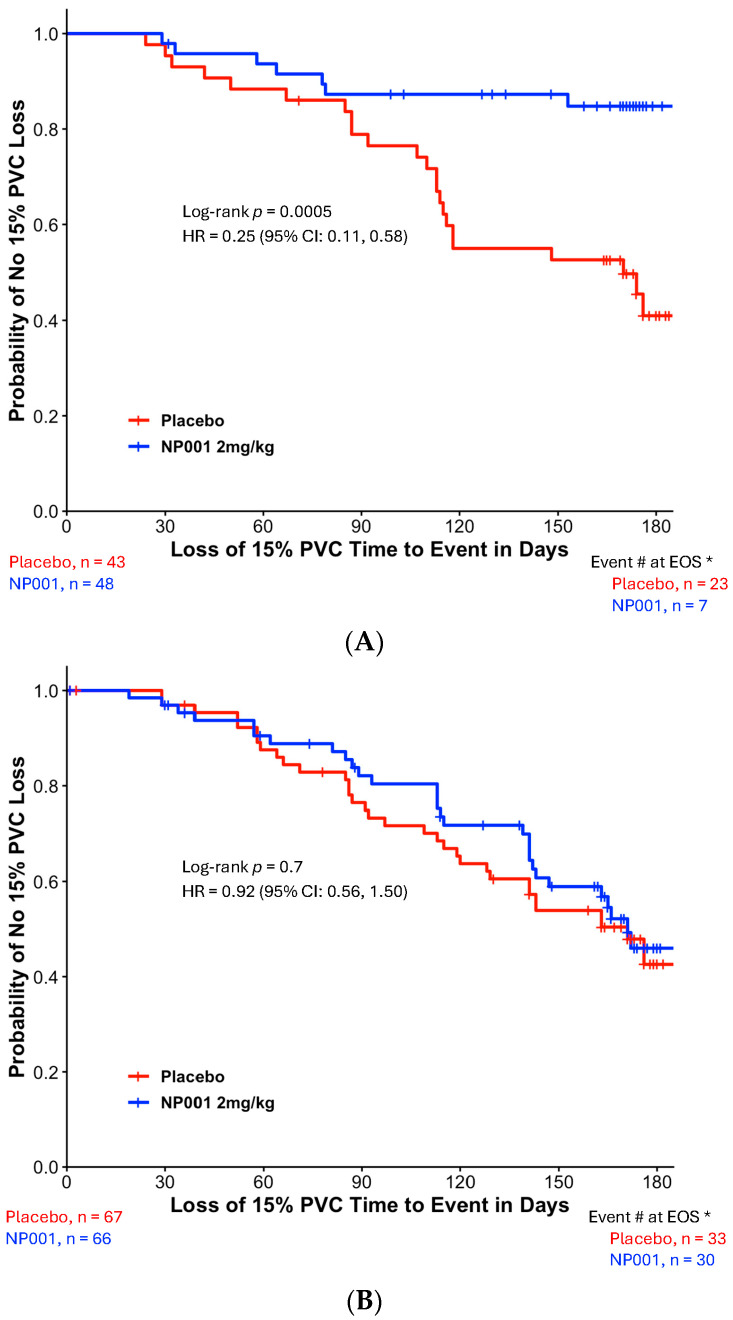
Kaplan–Meier plot of time to loss of 15% PVC by slowly and rapidly progressive ALS in NP001 phase 2 clinical trials. *, Event # at EOS: total number of patients that had events (PVC decline of ≥15%) by the end of the study (EOS). (**A**) Kaplan–Meier plot of time to loss of 15% PVC in patients with DPR < 0.50 ALSFRS-R score lost/month. ITT analysis in slowly progressive ALS with the 2 mg/kg NP001 group (blue, *n* = 48) and placebo (red, *n* = 43). Log-rank test, *p* = 0.0005; HR = 0.25, 95% CI: 0.11, 0.58. (**B**) Kaplan–Meier plot of time to loss of 15% PVC in patients with DPR ≥ 0.50 ALSFRS-R score lost/month. ITT analysis in rapidly progressive ALS with the 2 mg/kg NP001 subgroup (blue, n = 66) and placebo (red, *n* = 67). Log-rank test, *p* = 0.7; HR = 0.92, 95% CI: 0.56, 1.50.

Figure 2A,B show the TTE loss of 15% PVC in the DPR < 0.50 and DPR ≥ 0.50 groups, respectively. NP001-treated ALS patients with slowly progressive disease virtually stopped progression, whereas the placebo group progressed at a similar DPR as in the unseparated group in Figure 1 (Figure 2A, *p* = 0.0005). By the end of 6 months, 53% of placebos had progressed, whereas only 15% of those treated with NP001 had lost ≥ 15% of PVC. Table 2 shows the baseline demographics and ALS characteristics of the two treatment groups in the slowly progressive ALS population. No significant differences in baseline demographic and clinical characteristics were observed between the two treatment arms in the slowly progressive ALS group. The rapidly progressive patients were not significantly affected by NP001 vs. placebo, with both groups showing greater than 50% of patients experiencing loss of PVC events over the 6 months (Figure 2B). There were also no differences in the baseline demographic or clinical characteristics between the two treatment arms in the rapidly progressive ALS group.

### 3.3. Change in PVC from Baseline in ALS Patients Treated with NP001 vs. Placebo in Slow vs. Rapid Disease

**Figure 3:**
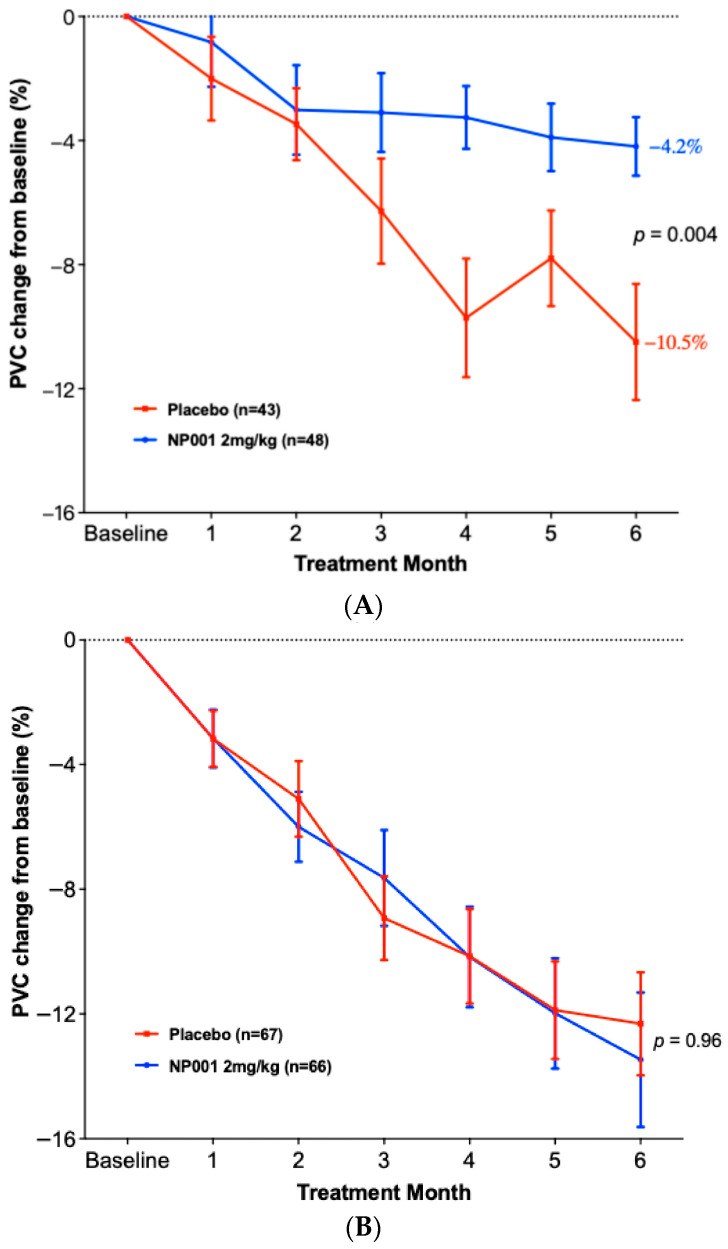
Comparison of PVC change from baseline by slowly and rapidly progressive ALS over the six-month NP001 phase 2 studies. (**A**) Participants with DPR < 0.50 ALSFRS-R score lost/month treated with NP001 experienced a slower PVC loss compared to placebo. PVC change from baseline for participants treated with NP001 (blue, *n* = 48) is compared to the placebo group (red, *n* = 43). Error bars represent the range, mean ± SEM (standard error of the mean) of PVC change from baseline. Average PVC lost over the 6 months of study: NP001: −4.2% (−0.7% per month); placebo: −10.5% (−1.75% per month). The NP001 treatment group showed a 60% slower PVC loss by the end of the study (Wilcoxon test, *p* = 0.004). (**B**) PVC change from baseline over the six-month study of participants treated with NP001 compared to placebo in rapidly progressive ALS (DPR ≥ 0.50 ALSFRS-R score lost/month). PVC change from baseline for participants treated with NP001 (blue, *n* = 66) compared to the placebo group (red, *n* = 67). Error bars represent the range, mean ± SEM of PVC change from baseline (Wilcoxon test, *p* = 0.96). No differences were seen between NP001 and placebo groups by the end of the study (NP001 = − 13.5% vs. Placebo = −12.3%) (Wilcoxon test, *p* = 0.96).

To test whether the TTE analysis would be related to a conventional measure of vital capacity loss over time, a change from baseline PVC analysis was performed on the slowly vs. rapidly progressive ALS patients. Figure 3A,B show the results of these analyses. As in Figure 2 and Figure 3, these show the selective sparing of loss in the change from baseline PVC in slowly (Figure 3A) but not rapidly progressive ALS patients (Figure 3B). Figure 3A shows that by the end of 6 months, NP001-treated, slowly progressive ALS patients had a 60% slowing in the loss of PVC from baseline (*p* = 0.004). Figure 3B shows that there was no difference in outcome between NP001 vs. placebo in rapid progressors.

### 3.4. Overall Survival Analysis in ALS Patients Treated with NP001 vs. Placebo in Slow vs. Rapid Disease

**Figure 4:**
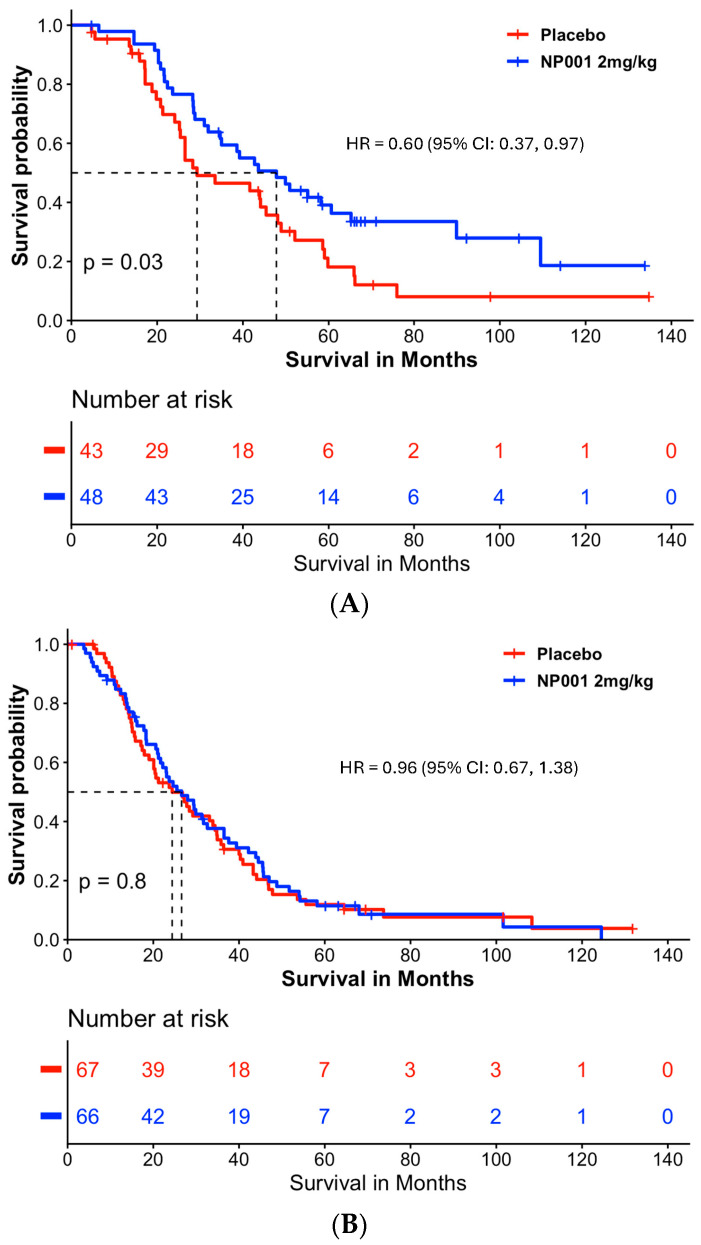
Survival analysis in slowly and rapidly progressive ALS in NP001 phase 2 clinical trials. (**A**) Kaplan–Meier curve of survival probability for patients with slowly progressive ALS (DPR < 0.50 ALSFRS-R score lost/month).The median survival over the entire follow-up duration was 47.8 months (95% CI: 34.7, 89.9) and 29.3 months (95% CI: 25.4, 48.9) in the 2 mg/kg NP001 (blue) and placebo (red) groups, respectively (log-rank, *p* = 0.03). Patients treated with NP001 had a 40% lower risk of death than those on placebo: HR = 0.60 (95% CI: 0.37, 0.97) in patients with slow DPR at baseline. (**B**) Kaplan–Meier curve of survival probability for patients with rapidly progressive ALS (DPR ≥ 0.50 ALSFRS-R score lost/month) from NP001 Phase 2 Trials. The median survival over the entire follow-up duration was 26.6 months (95% CI: 21.7, 36.5) and 24.4 months (95% CI: 20.1, 34.8) in the 2 mg/kg NP001 (blue) and placebo (red) groups, respectively (log-rank, *p* = 0.8). No difference in risk of death was observed between patients treated with NP001 and placebo controls: HR = 0.96 (95% CI: 0.67, 1.38).

Figure 4A shows that NP001-treated patients had an extension of median survival of 18.5 months (47.8 vs. 29.3) in the slowly progressive ALS group (Log-rank *p* = 0.03; HR = 0.60, 95% CI: 0.37, 0.97) but this was not significantly different from placebo in the rapidly progressive group (Figure 4B, median survival in the NP001 treated was 26.6 months vs. 24.4 months for placebo). Importantly, the placebos in the slowly progressive group had a median OS of 29.3 months as compared to 24.4 months in the rapidly progressive group, values insignificant by comparison to the 47.8 months experienced by NP001 recipients.

## 4. Discussion

ALS is a heterogeneous disease; however, the majority of ALS patients die from a progressive loss of respiratory function. In the current study, the focus on respiratory function in the context of drug development of disease-modifying therapeutics for ALS allows the use of an objective measurement tool, VC (SVC and FVC), rather than reliance on the subjective ALSFRS-R scoring system to test for efficacy. Measurements of VC in the evaluation of ALS disease progression have been linked to overall survival [12], supporting the use of this evaluation tool as described, using the time to event of the loss of 15% PVC, to test drugs that are potentially disease-modifying.

NP001 is an intravenous formulation of sodium chlorite, a prodrug for the dominant regulator of innate immune activation, taurine chloramine [2,8,10,26]. Extensive in vitro studies have confirmed that chlorite is converted to hypochlorite in the presence of heme-associated iron through a Michaelis-Menten reaction [27] and that, upon conversion to TauCl within macrophages, it down-regulates the expression of NF-kB [3,4] and induces the expression of the macrophage suppressive factor, Heme oxygenase-1 [6]. Sodium chlorite was a major component of WF10, a drug mixture approved in Thailand for patients with irradiation-associated bleeding syndromes [28,29]. Through extensive testing of WF10 in post-radiation syndrome patients, the dose of 2 mg/kg and a frequency of every three to four weeks was defined as optimal for this endarteritis-like disease. In patients with post-radiation hemorrhagic bleeding from the bladder, after response to the drug, more than half of the patients did not require further therapy after 2 five-day cycles of WF10 three weeks apart [28]. Further studies of WF10 in patients with advanced AIDS also confirmed the intermittent dosing regimen and documented the regulation of monocyte expression of activation antigens and TNF-α RNA after a single 5-day cycle of the drug [27]. Although WF10 was not a hemolytic agent [27], chronic wound healing and post-radiation syndrome patients suffered high rates of anemia [28,29]. Given the absence of anemia observed in ALS patients repeatedly exposed to NP001 [8], it is possible that WF10’s other anions, such as chlorate, may have contributed to the anemia [30].

The data used for the evaluation of the TTE tool in ALS were derived from two double blind placebo-controlled 6-month clinical trials testing the immune regulator molecule, NP001, as compared to placebo. The two phase 2 NP001 trials were performed using ALS patients within 3 years of symptom onset, with phase 2A having a requirement that participants have a baseline FVC > 70%. The results of this study suggested that participants with > than the median level of plasma C reactive protein (CRP), 1.13 mg/L, showed a trend of efficacy in change from baseline levels of ALSFRS-R scores [8]. The phase 2B included only participants with plasma CRP levels above 1.13 mg/L, but allowed the inclusion of participants with SVC levels as low as 65% in the three years since symptom onset [9]. Neither trial alone showed significant efficacy using the ALSFRS-R score change from baseline. When combined in a post hoc analysis, the subset between 40 and 65 years old with a CRP > 1.13 mg/L had a loss of VC slowing of 51%. The VC result and a change from baseline in ALSFRS-R score were significantly different in treated patients as compared to placebos in this post hoc analysis [9]. As both of these trials were completed in 2011 (phase 2A) and 2017 (phase 2B), long-term survival data were available. In an ITT analysis, the NP001 treatment for 6 months showed a 4.8-month survival advantage, and in the ≤ 65-year-old group, there was a 10.8-month advantage [13]. The lack of a correlation between the ITT survival data and the ALSFRS-R score change from baseline observed in these trials suggested that another evaluation be undertaken, one that tracked the evolution of the major cause of death, respiratory failure.

The data analyzed in the current study included the entire ITT population of patients from the two trials evaluated combined, as well as in individual trial datasets. No further selection criteria, such as CRP or age, were employed in the analyses reported in this manuscript. In the current study, a loss of 15% of predicted VC was chosen as the TTE biomarker threshold based on studies by Andrews [12] showing that the loss of PVC in the majority of ALS patients was 2.5% per month or 15% over 6 months. The endpoint (PVC change ≥ 15%) in the ITT population showed a statistically significant risk reduction of 40% for NP001 compared to placebo.

The initial association between NP001 responsiveness and levels of plasma CRP suggested an association between inflammation, ALS pathogenesis, and responsiveness to innate immune regulation with NP001. A major Swedish ALS natural history study [20] suggested another association between plasma CRP levels and ALS pathogenesis. In this study, ALS patients were matched up with 5× normal age-matched controls, and their levels of serum creatinine were evaluated over time in parallel to their levels of CRP and time before and after diagnosis with ALS. In patients who developed ALS, serum creatinine began dropping 2 years pre-diagnosis and continued to drop as ALS progressed. In parallel measurements, CRP levels became elevated over the controls beginning 1–2 years after diagnosis. Thus, ALS patients whose symptom onset was early and diagnosis was late would be those with slightly elevated CRP who are potentially responsive to NP001. So, rather than just having ALS with inflammation, could there be a form of ALS that is slowly progressive and driven by an immunopathogenic process, potentially amenable to regulation with NP001, rather than inflammation being secondary to the death of tissues related to ALS?

To test whether the rate of disease might be both related to loss of respiratory function as well as responsiveness to NP001, a value of disease progression rate was employed to separate slow from rapid progressors. The loss of 0.50 units or less of the ALSFRS-R score per month has been used by recent studies [31] as defining slowly progressive ALS (slow ALS). This cut point divided the ITT population of patients studied in the two phase 2 NP001 trials so that 41% were designated as slow and the remainder as rapidly progressive ALS. As Figure 2, Figure 3 and Figure 4 show, the majority of disease regulation activity was in the slowly progressive group. Even though these are post hoc studies, the *p* values of 0.0005 and 0.004 in Figure 2 and Figure 3, vital capacity-sparing activities are so small that statistically, the results are highly unlikely to be related to chance. Especially so, given the results of the TTE PVC measurement result tracking, which are absolutely in parallel to the standard VC change from baseline shown in Figure 2 and Figure 3. Most importantly, the changes in VC related to NP001 administration were associated with an extension of median survival of 18.5 months over placebo. Given the consistent VC and OS changes associated with innate immune regulation of slowly progressive vs. rapidly progressive ALS, it is possible that this major subset of ALS may be effectively targeted as an immunopathogenic process.

## 5. Conclusions

NP001 is mechanistically relevant for the treatment of inflammation in association with ALS pathogenesis. The association of 15% PVC loss with TTE outcomes in both the combination dataset as well as each individual clinical trial dataset speaks to the consistency of the metric and its association with survival. The further linkage between the TTE analysis of ALS patients with slowly vs. rapidly progressive disease, with a conventional loss from baseline in PVC and survival in ALS patients, links three separate outcomes with clinically significant responses to NP001. Whether NP001 responsiveness is related to an immunopathogenic form of ALS has yet to be determined. We propose that response to NP001 in separate measures of respiratory function tied to survival provides a rational basis for the performance of a phase 3 trial linking the change in VC with measures of overall function such as in the ALSFRS-R score.

As with all post hoc analyses, there is a risk for a Type 1 error. A longer confirmatory prospective multi-center randomized placebo-controlled double-blinded study of NP001 examining changes in measures of VC, along with relevant clinical outcome associations, is warranted.

## Figures and Tables

**Table 1:** Baseline demographics and characteristics of participants with slowly and rapidly progressive ALS ^1^ in intent-to-treat (ITT) population ^2^ from NP001 Phase 2 clinical trials.

	DPR < 0.50	DPR ≥ 0.50	
Characteristics	(*n* = 91)	(*n* = 133)	*p* Value
Sex, *n* (%)			1.0
Female	29 (31.9%)	43 (32.3%)	
Male	62 (68.1%)	90 (67.7%)	
Age at baseline (years), mean ± SD	56.1 ± 10.1	55.7 ± 10.9	0.87
Site of ALS onset, *n* (%)			0.70
Bulbar	14 (15.4%)	18 (13.5%)	
Limb	77 (84.6%)	115 (86.5%)	
El Escorial criteria for ALS, *n* (%)			NS
Definite	39 (42.9%)	57 (42.9%)	
Possible	9 (9.9%)	8 (6.0%)	
Probable	38 (41.8%)	59 (44.4%)	
Probable Laboratory Supported	5 (5.5%)	9 (6.8%)	
Concurrent riluzole use, *n* (%)			0.15
Yes	56 (61.5%)	95 (71.4%)	
No	35 (38.5%)	38 (28.6%)	
ALSFRS-R score at baseline, mean ± SD	40.6 ± 3.6	35.4 ± 5.3	<0.0001
PVC ^3^ at baseline (%), mean ± SD	89.7 ± 15.9	85.3 ± 15.5	0.08
Duration of ALS symptom (months), mean ± SD	22.79 ± 7.68	14.91 ± 7.15	<0.0001
DPR ^4^ at baseline (ALSFRS-R score lost/month)	0.33 ± 0.12	1.03 ± 0.89	<0.0001
CRP at baseline (mg/L), mean ± SD	3.62 ± 3.59	3.48 ± 4.86	0.65

Abbreviation: *n*, number of participants. NS, not significant. SD, standard deviation. ^1^ Slowly and rapidly progressive ALS: baseline DPR < 0.50 units of ALSFRS-R score lost/month was defined as slowly progressive ALS, and rapidly progressive ALS were those whose DPR ≥ 0.50 units of ALSFRS-R score lost/month at baseline. ^2^ Intend-to-treat (ITT) population, all randomized subjects who received at least one dose of study infusion. ^3^ PVC, Predict vital capacity. ^4^ DPR, ALS disease progression rate defined as units of ALSFRS-R score lost per month.

**Table 2:** Baseline demographics and characteristics of the intent-to-treat (ITT) population ^1^ with slowly progressive ALS ^2^.

	NP001 2 mg/kg	Placebo	
Characteristics	(*n* = 48)	(*n* = 43)	*p* Value
Sex, *n* (%)			0.26
Female	18 (37.5%)	11 (25.6%)	
Male	30 (62.5%)	32 (74.4%)	
Age at baseline (years), mean ± SD	56.1 ± 9.4	56.0 ± 11.0	0.73
Site of ALS onset, *n* (%)			0.56
Bulbar	6 (12.5%)	8 (18.6%)	
Limb	42 (87.5%)	35 (81.4%)	
El Escorial criteria for ALS, *n* (%)			NS
Definite	18 (37.5%)	21 (48.8%)	
Possible	6 (12.5%)	3 (7.0%)	
Probable	21 (43.8%)	17 (39.5%)	
Probable Laboratory Supported	3 (6.3%)	2 (4.7%)	
Concurrent riluzole use, *n* (%)			1.0
Yes	30 (62.5%)	26 (60.5%)	
No	18 (37.5%)	17 (39.5%)	
ALSFRS-R score at baseline, mean ± SD	40.2 ± 4.1	41.0 ± 3.0	0.54
PVC ^3^ at baseline (%), mean ± SD	90.5 ± 16.8	88.7 ± 15.1	0.49
Duration of ALS symptom (months), mean ± SD	23.67 ± 7.84	21.79 ± 7.47	0.28
DPR ^4^ at baseline (ALSFRS-R score lost/month)	0.33 ± 0.12	0.33 ± 0.11	1.0
CRP at baseline (mg/L), mean ± SD	4.05 ± 4.20	3.17 ± 2.80	0.30

Abbreviation: *n*, number of participants. NS, not significant. SD, standard deviation. ^1^ Intent-to-treat (ITT) population, all randomized subjects who received at least one dose of study infusion. ^2,4^ Slowly progressive ALS, participants with baseline DPR (disease progression rate) < 0.50 units of ALSFRS-R score lost/month. ^3^ PVC, Predict vital capacity.

## Data Availability

The original contributions presented in the study are included in the article, further inquiries can be directed to the corresponding author.

## Abbreviations

The following abbreviations are used in this manuscript:

ALS	Amyotrophic Lateral Sclerosis
ALSFRS-R	Revised ALS Functional Rating Scale Score
DPR	Disease Progression Rate
EOS	End of Study
FDA	Food and Drug Administration
FVC	Forced Vital Capacity
HR	Hazard Ratio
IPF	Idiopathic Pulmonary Fibrosis
ITT	Intention-to-Treat
NF-kB	Nuclear Factor-kappa B
OS	Overall Survival
PVC	Predicted Vital Capacity
SEM	Standard Error of the Mean
SD	Standard Deviation
SOD1	Superoxide Dismutase-1
SVC	Slow Vital Capacity
TauCl	Taurine Chloramine
TNF-α	Tumor Necrosis Factor alpha
TTE	Time to Event
VC	Vital Capacity
